# Barriers and enablers to health care providers assessment and treatment of knee osteoarthritis in persons with type 2 diabetes mellitus: A qualitative study using the Theoretical Domains Framework

**DOI:** 10.1016/j.ocarto.2022.100299

**Published:** 2022-08-07

**Authors:** Lauren K. King, Owen Krystia, Esther J. Waugh, Crystal MacKay, Ian Stanaitis, Jane Stretton, Alanna Weisman, Noah M. Ivers, Janet A. Parsons, Lorraine Lipscombe, Gillian A. Hawker

**Affiliations:** a Department of Medicine, University of Toronto, Toronto, ON, Canada; b Women's College Research Institute, Women's College Hospital, Toronto, ON, Canada; c Department of Physical Therapy, University of Toronto, Toronto, ON, Canada; d Patient Research Partner, Toronto, ON, Canada; e Lunenfeld-Tanenbaum Research Institute, Mount Sinai Hospital, Toronto, ON, Canada; f Department of Community and Family Medicine, University of Toronto, Toronto, ON, Canada; g Applied Health Research Centre and Li Ka Shing Knowledge Institute, St. Michael's Hospital – Unity Health Toronto, Toronto, ON, Canada

**Keywords:** Osteoarthritis, Type 2 diabetes mellitus, Qualitative, Theoretical domains framework

## Abstract

**Objectives:**

Symptomatic knee osteoarthritis (OA) commonly co-exists in persons with Type 2 diabetes (T2DM) and may impede diabetes self-management. Yet, OA is often underdiagnosed and undertreated due to competing health care demands. We sought to determine healthcare providers' (HCPs’) perceptions of the barriers and enablers to assessing and treating knee OA in persons with T2DM.

**Design:**

We conducted 18 semi-structured telephone interviews with HCPs who manage persons with T2DM (family physicians, endocrinologists, diabetes educators). Interviews were analyzed deductively using Theoretical Domains Framework (TDF), a framework developed to comprehensively identify behavioural determinants. Within relevant domains, data were thematically analyzed to generate belief statements, and these were compared across the different HCP disciplines.

**Results:**

Six TDF domains influenced HCPs behaviour to assess and treat knee OA in persons with T2DM. For all HCPs, important barriers included not seeing assessment/treatment of joint pain as a priority for their patients (intention), and insufficient access to required resources such as physiotherapy to treat OA (environmental context and resources). Endocrinologists and diabetes educators perceived having insufficient knowledge and skills to identify and manage OA (knowledge, skills), did not consider it within their professional role to do so (professional role and identity), and perceived other physicians would not want to receive a referral for OA care (social influences).

**Conclusions:**

We identified barriers and enablers encountered by diabetes HCPs to assessing and treating knee OA in persons with T2DM involving multiple domains of the TDF. These will help inform development of a complex intervention to improve health outcomes.

## 1 Introduction

Despite recent advances in clinical care, the prevalence of type 2 diabetes mellitus (T2DM) continues to rise [1] and there has been a recent resurgence in rates of diabetes complications [2]. Multimorbidity, the presence of two or more chronic conditions, is also increasing [3]. Osteoarthritis (OA), characterized by chronic joint pain and functional impairments [4], is one of the most common conditions to cluster with T2DM [5,6] due to shared risk factors, such as obesity and older age [7]. Approximately 17% of individuals with T2DM have knee OA [8], which is responsible for the majority of OA-related disability globally [1]. The combination of T2DM and knee OA has the potential for deleterious effects. While guidelines recommend that persons with T2DM engage in 150 ​min of moderate-to-vigourous physical activity per week to improve disease outcomes [9,10], walking difficulty [11] and other sequelae of chronic, painful knee OA, such as depression and fatigue [12,13], may make T2DM self-management more challenging [14]. Prior research demonstrates that individuals with T2DM and chronic pain are less engaged in diabetes self-management [15], while those with knee OA-related walking difficulty are at increased risk of cardiovascular events and other diabetes-specific complications [16]. Despite this, first-line OA therapies recommended to manage OA pain and disability [17] (including disease education, physical activity/exercise, and appropriate pharmacotherapy) remain underutilized [[18], [19], [20]]. Given the competing demands of T2DM management [21], persons with T2DM may receive even less attention to their OA by their health care providers (HCPs). As adequate treatment of OA symptoms can have direct benefits on T2DM self-management, there is an urgent need to improve the implementation of evidence-based knee OA care in persons with T2DM to improve both T2DM and OA outcomes.

Our overarching research goal is to develop a theory-informed complex intervention to improve the assessment, diagnosis, and evidence-based treatment of symptomatic knee OA in persons with T2DM to improve health outcomes. The UK Medical Research Council (MRC) guidance on the development of complex interventions recommends using theory to first understand the determinants of the health behaviour(s) [22,23]. Thus, within the first phase of intervention development, as part of engaging multiple stakeholder groups relevant to the care of persons with T2DM, the current study, focusing on one stakeholder group, sought to understand diabetes HCPs’ perceptions of the barriers and enablers to assessing and treating knee OA in persons with T2DM, using the Theoretical Domains Framework (TDF) [24,25].

## 2 Methods

In this study, informed by qualitative description [26], we conducted semi-structured interviews with a sample of HCPs who manage patients living with T2DM across different disciplines and practice locations in Ontario, Canada. Qualitative description is seen as an important methodology for describing poorly-understood healthcare phenomena, including when information is being used to develop an intervention, and allows for flexibility of methods [27]. Interviews were completed in September and October 2020. We followed guidance by Atkins et al. [25] on the collection and analysis of qualitative data using the TDF, an implementation science framework that incorporates a range of theoretical constructs to comprehensively identify determinants of behaviour [24]. We provide further details on the TDF domains and definitions used in Supplementary Table A. These include incorporating TDF domains in the interview guide; data coding to the TDF domains; generating themes/belief statements within each domain relevant to the behaviour of interest; and presenting findings framed by the TDF. We completed this study alongside assessments of other stakeholder groups that will be published separately.

This study was approved by the Research Ethics Review Boards at Women's College Hospital and at the University of Toronto. We followed the Consolidated Criteria for Reporting Qualitative Studies (COREQ) guidelines for reporting qualitative research [28].

### 2.1 Study setting

In Ontario, Canada, individuals with chronic conditions, such as T2DM, present to primary care providers (family physicians or nurse practitioners) as the first point of contact in the health care system. A referral from a primary care provider or other physician is needed for an individual to be seen by a medical specialist (e.g., endocrinologist who provides specialized diabetes care). Additionally, individuals with T2DM may receive concurrent care for self-management by certified diabetes educators (often nurses or dietitians) in diabetes education centres. The health care system in Ontario is publicly funded and privately administered. The Ontario Health Insurance Plan provides coverage for most medical and emergency services provided in Ontario. However, it does not provide universal financial coverage. Of particular relevance to this study, prescription drugs and physiotherapy for those who are not on social assistance and/or under age 65 are paid for out-of-pocket by patients.

### 2.2 Sampling

We recruited participants through the networks of our research team. Via e-mail, we invited HCPs who practiced as primary care providers (family physician or nurse practitioner), endocrinologists or diabetes educators in Ontario, Canada to participate. We used a purposeful sampling strategy to ensure variability in years of experience, gender, and to include HCPs from a range of practice locations. We stopped data collection at the point when data did not add new insights into our research question [29]. Prior to conducting the interview, participants provided verbal and written consent.

### 2.3 Data collection

We developed a semi-structured interview guide that aimed to comprehensively uncover determinants of HCP behaviour according to the TDF, a framework validated for use in implementation science research [30]. We have included our interview guide in Supplementary Table B. The behaviours of interest for the current study were assessing and treating knee OA in persons with T2DM. We defined these behaviours as in any way addressing OA through history, physical exam, investigations, advice, prescriptions or referral to another provider. Participants were also invited to speak about any aspect of the topics raised that they saw as relevant. At the end of the interviews, participants were asked their age range, gender, and years in clinical practice.

One member of the research team, a rheumatologist and doctoral trainee (LK), conducted all semi-structured telephone interviews and had no pre-existing relationships with participants other than with one participant in a research capacity. Interviews lasted 45–60 ​min, and were audio-recorded, and the recordings were professionally transcribed verbatim and anonymized prior to analysis. Data were organized using NVivo 12 software (QSR International Pty Ltd).

### 2.4 Data analysis

Interview transcripts were analyzed using content analysis [31], applying TDF domains in a deductive manner [25]. All TDF domains were considered. Two researchers (OK, LK) initially coded four transcripts in duplicate and met to compare and discuss coding decisions. When coding disagreement occurred, areas of difference were resolved through discussion and review of original transcripts. Once there was agreement on how to apply codes, one researcher (OK) coded all subsequent transcripts.

We then conducted thematic analysis [32] of data coded within each TDF domain, to inductively develop themes in the form of belief statements [25]. Belief statements reflected specific determinants of diabetes HCPs assessing and/or treating knee OA in persons with T2DM that we identified as prominent across interviews. Belief statements were developed by two researchers (OK, LK) and were reviewed with a senior mixed methods researcher (GH). We contrasted findings across HCP disciplines. In addition to following guidance by Atkins et al. [25], rigour was enhanced through use of multiple analysts, analytic memos, and a research audit trail [33,34].

## 3 Results

We interviewed 18 HCPs (8 endocrinologists, 7 primary care providers [6 family physicians, 1 primary care nurse practitioner], 3 diabetes educators), of whom 14 were women (77.8%), 12 were less than 50 years old (66.7%), 5 (27.8%) had fewer than 10 years in clinical practice, the majority practiced in urban locations (94.4%), and were from eight different practice locations (Table 1).

**Table 1:** Health care provider characteristics (n ​= ​18).

Characteristic	n (%)
Gender, woman	14 (77.8)
Age	
30–39 years	5 (27.8)
40–49 years	7 (38.8)
50–59 years	3 (16.7)
70–79 years	3 (16.7)
Profession	
Endocrinologist	8 (44.4)
Family Physician/Primary care Nurse Practitioner	7 (38.9)
Diabetes Educator (NP, Clinical Nurse Specialist)	3 (16.7)
Years in Practice	
0–9 years	5 (27.8)
10–19 years	9 (50.0)
20–29 years	2 (11.1)
≥30 years	2 (11.1)
Urban practice location	17 (94.4)

We identified six TDF domains as relevant to the HCP behaviour of assessing and/or treating knee OA in persons with T2DM and present inductively developed belief statement(s) for these domains (Table 2). We present findings with illustrative quotations. We also indicate where there were differences in results observed between endocrinologists/diabetes educators and family physicians.

**Table 2:** Theoretical Domains Framework analysis with inductively developed belief statements.

TDF Domain	Belief Statements and Sample Illustrative Quote	
	Endocrinologists and Diabetes Educators	Primary Care Providers
Knowledge	I lack sufficient knowledge about OA to diagnose or make treatment recommendations *“And in terms of the latest treatments in arthritis care, I'm not fully up to date on that literature …” P2 (endocrinologist)* *“I don't even remember getting any education on osteoarthritis.” P12 (diabetes educator)*	–
Skills	I am not comfortable performing a joint assessment *“We have never received special training of this nature.”* P6 *(diabetes educator)* *“It's hard to tackle [joint assessment] in great detail, especially when it's not something that we necessarily have tons of direct training on.” P9 (endocrinologist)*	I am comfortable performing a joint assessment *“Well, I've been in practice quite a long time, and working in Emerg. I feel quite comfortable doing a joint pain assessment, deciding whether or not imaging is going to help or make a difference in what the management plan is going to be so I'm not insecure in my MSK part of medicine.” P15*
Professional role and identity	I do not see OA as a comorbidity that diabetes specialists manage *“I feel like [joint pain] doesn't [fit into my professional role]. I'm going to be perfectly honest.” P8 (endocrinologist)*	While the primary care providers' role includes assessment of joint pain, other health professionals are needed to support OA care *“If we had access to, not an exercise physiologist, but even a physio that could spend the time to go through with someone, to really sit down and figure out what they have access to, what they're willing to do, all that sort of stuff, to come up with a reasonable plan with them that they'll actually do, and follow up with them. That would be really great.” P10*
Intentions	I prioritize assessment of diabetes control and complications and do not seek to address joint pain *“Everything is focused on the A1C. Get an A1C every three months, see your doctor, whether it's a family doctor or an endocrinologist for your diabetes visit every three months. There's just too much to cover in a 15-min visit every three months.” P8 (endocrinologist)*	I prioritize assessment of diabetes control and complications and do not seek to address joint pain *“In my mind, we probably get to the osteoarthritis last, and that would mean that there's less time.” P16*
Environmental context and resources	There are insufficient resources available within the health care system for OA care, including access to physical therapy *“I don't have a good system to send them to physical therapy or anything else from an Allied Health perspective. I wish I did actually.” P7 (endocrinologist)*	There are insufficient resources available within the health care system for OA care, including access to physical therapy *“Patients who are well off enough or who have extended health coverage, that's great. They can be referred for physio, but then there's that big silo of working-class people who don't have $80.00 twice a week to pay for physiotherapy. I think that is a difficult situation for family doctors. If someone really does need intensive physical therapy, what do we do?” P15*
Social influences	I perceive rheumatologists do not want to receive a referral for OA management *“I'll give another comment in terms of patients with joint pain, and again maybe I have the wrong sense, but as an endocrinologist my general sense is that rheumatologists don't love to see patients with bread-and-butter osteoarthritis.” P9 (endocrinologist)*	–

### 3.1 Domain: knowledge

Belief statement: I lack sufficient knowledge about OA to diagnose or make treatment recommendations (endocrinologist, diabetes educator).

Participants who were endocrinologists and diabetes educators described lack of sufficient knowledge about OA, which limited their ability to identify or make treatment recommendations. For example, one endocrinologist discussed how they were not up to date on OA literature and did not know the current treatment guidelines for arthritis care. Another endocrinologist reflected that they were limited to making only basic treatment recommendations because they had never completed any formal arthritis training.

“I wouldn't say that I ever did any formal training to tell me what's good and what's bad for people who've got OA, but I'll make some of those very basic recommendations.” P8 (endocrinologist).

“I wouldn't recommend therapy. I don't feel comfortable with that.” P5 (endocrinologist).

Diabetes educators specifically described having received no formal education on arthritis during their health professional training, and thus they were not comfortable discussing OA care with their T2DM patients.

“I don't even remember getting any education on osteoarthritis.” P12 (diabetes educator)

In contrast, participants who were primary care providers generally felt their knowledge regarding OA was adequate to facilitate diagnosis and initiation of treatment.

### 3.2 Domain: skills

Belief statement: I am not comfortable performing a joint assessment (endocrinologist, diabetes educator).

Participants who were endocrinologists spoke about their discomfort performing a musculoskeletal exam, other than for specific musculoskeletal manifestations of diabetes (e.g., finger flexor tenosynovitis). While they had learned to perform a joint examination in medical school and in their internal medicine training, these skills were not something they had sufficiently maintained in their endocrinology practice. Thus, this lack of practice and maintenance of skills was seen as a barrier to being able confirm a diagnosis of knee OA in someone reporting joint symptoms.

“I haven't examined joints since my training, so I don't feel that I'm well trained to do a joint exam even anymore and sort of lean to interpret or diagnose a problem.” P1 (endocrinologist).

Lack of any training in musculoskeletal assessment was expressed by diabetes educators as a barrier to evaluating joint pain.

“I certainly don't feel confident in assessing someone's joint … it's definitely not something I would deal with now.” P12 (diabetes educator).

Belief statement: I am comfortable performing a joint assessment (primary care provider).

In contrast, participants who were family physicians expressed confidence in their joint examination skills and this was seen to facilitate assessment of OA. Musculoskeletal issues were more regularly encountered in their clinical practices, resulting in familiarity with the joint examination and identification of OA.

“Yeah, I feel comfortable assessing joint pain. I would say the majority of what we see is more transient things, rather than joint pain coming from either a serious rheumatic condition or a serious OA of the hip that needs surgery. Most of the time it's more aches and pains, and my back hurts, and I've been sitting in a position too long, it's sore when I get up, it's more those kinds of things. Although we obviously see some of the more serious stuff, but that, I would say, is less common.” P10 (primary care provider).

### 3.3 Domain: professional role and identity

Belief statement: I do not see OA as a comorbidity that diabetes specialists manage (endocrinologist, diabetes educator).

We heard from participants who were endocrinologists and diabetes educators that they did not view knee OA as a diabetes-specific comorbidity that was within their scope of care.

“I think most of my patients, they know that the way we run this is a very focused practice, very much the metabolic and cardiovascular implications of diabetes is what we focus on.” P4 (endocrinologist).

While many recognized the impact of chronic joint pain on physical activity, weight management and mood, they did not see that it was their role to address it. They would frequently redirect patients reporting joint symptoms to see their primary care provider, as they saw addressing symptoms of OA as fitting best to that professional role.

“So, generally I would refer them first back to their family doctor or primary care provider for an assessment. I feel like it's sort of out of the scope of my expertise as a specialist.” P1 (endocrinologist).

“I always tell them their first stop should be their family doctor.” P4 (endocrinologist).

Belief statement: While the primary care providers’ role includes assessment of joint pain, other health professionals are needed to support OA care (primary care provider).

In contrast, primary care providers saw that identifying knee OA and suggesting management strategies was within their professional scope. However, the exception was physical activity for OA, where many primary care providers viewed it was outside their role to prescribe and monitor physical activity, given the time and perceived skill needed to do so.

“It takes time to do all that stuff, and you know what, I'm probably not the right person in the system, to be spending … I don't have the time to spend tons of time going through all these exercise options, and following up, and two weeks later, calling them to say, hey, how did you do with your program.” P10 (primary care provider).

Primary care providers relied on other health professionals to assist in delivering OA care and saw the need for a system in place to facilitate comprehensive OA management outside of primary care offices.

“So, what we have is a totally fragmented system, and maybe it’s fine to have a fragmented system for some things that are super-rare where you can get the one person that has the rare thing to where they need to go. But for something that is so common, osteoarthritis, why do we not have a pathway or a system?” P16 (primary care provider).

One participant described regularly providing comprehensive physical activity advice to patients, including those with OA; they endorsed personally engaging in regular physical activity and appreciating its benefits.

### 3.4 Domain: intentions

Belief statement: I prioritize assessment of diabetes control and complications, and do not seek to address joint pain (endocrinologist, diabetes educator, primary care provider).

Participants from all disciplines indicated that their priorities for patients with T2DM were diabetes glucose control and monitoring of diabetes-specific complications. They emphasized that a diabetes visit has many elements to cover and that frequently left little or no time to address additional comorbidities, such as joint pain. Participants would generally not initiate a discussion about joint symptoms.

“I don't ask, now that I think of it, I don't ask them [about joint pain] …” P17 (primary care provider).

Screening or assessment of OA was not considered of sufficient priority to be included as part of the diabetes assessment. If brought up by patients during an encounter, often heard as a limitation to physical activity, endocrinologists and diabetes educators would ask patients to discuss with their primary care provider.

“I generally wouldn't get too involved in trying to diagnose it or manage it [joint pain]. I would say for most patients, if I hear about it, it's usually as a barrier to physical activity and how that's negatively impacting their weight and their blood sugars.” P1 (endocrinologist).

Several participants described how they avoided specifically asking about joint pain as they might uncover a new issue that they would have to deal with. They viewed pain as a complex topic which had potential to take over their diabetes appointment.

“I wonder if part of that is that, in all honesty, if you were to ask [about joint pain], that could open up another whole other can of worms for patients, and then it could take over the whole appointment potentially.” P12 (diabetes educator).

### 3.5 Domain: environmental context and resources

Belief statement: There are insufficient resources available within the health care system for OA care, including access to physical therapy (endocrinologist, diabetes educator, primary care provider).

All participants described lack of resources within the health care system as a key barrier to providing care for OA; even when participants saw an opportunity to provide OA care, they perceived they had few options. Participants particularly recognized that for many of their patients, referring to physiotherapy was not an option due to significant patient out-of-pocket costs, leaving them unsure what more they could do for their patients.

“I would acknowledge that it’s sometimes a hard area to deal with and often requires resources that are not necessarily always readily available within the publicly funded system” P9 (endocrinologist).

When referring to other physicians, there were often long wait times that made it difficult to provide care to the symptomatic patient at the time they most needed it. Many participants, across disciplines, expressed a desire for a government-funded program to refer patients with suspected or confirmed OA where they could receive expert assessment and longitudinal follow up. Participants expressed that if resources for OA care were available and readily accessible, they might be more inclined to invite patients to discuss joint pain.

**“**Because sometimes the wait to be seen by somebody who does the injections, be it a rheumatologist or an orthopedic surgeon, can be really quite delayed and the patient is already coming to me with problems, so, to have them wait is really frustrating. P17 (primary care provider).

“I don't know, I almost wish that there was a rheumatology clinic that will see them within two weeks for that kind of thing because just where we've done all the baseline stuff but they're still struggling with their symptoms.” P11 (primary care provider).

### 3.6 Domain: social influences

Belief statement: I perceive rheumatologists do not want to receive a referral for OA management (endocrinologist).

One endocrinologist perceived rheumatologists would not want “bread-and-butter” OA referrals and had a negative experience when they had done so in the past. Thus, the perception of OA being an undesired reason for referral limited referral behaviour. These data came from a single participant, however given its potential importance, we have included them.

“I'll give another comment in terms of patients with joint pain, and again maybe I have the wrong sense, but as an endocrinologist my general sense is that rheumatologists don't love to see patients with bread-and-butter osteoarthritis.” P9 (endocrinologist).

## 4 Discussion

In this qualitative study, we identified perceived barriers and enablers of HCPs with respect to assessing and treating knee OA in persons with T2DM across six domains of the TDF. We focused on this population because of the sequelae of underdiagnosed and undertreated knee OA on diabetes self-management [15] and risk of diabetes complications [16]. Participants from all disciplines expressed that addressing OA was a low priority relative to other elements of T2DM care (intentions) and perceived insufficient resources available to allow them to provide care for OA (environmental context and resources). Barriers specifically perceived by endocrinologists and diabetes educators included insufficient knowledge about OA that limited confidence in assessing joint pain, performing a joint examination, or in making treatment recommendations (knowledge, skills), that OA was out of their scope of care (professional role and identity), and that other specialists would not want a referral for OA care (social influences). This builds on prior research examining barriers to OA management among primary care providers [21,35] to include the challenge relating to multimorbidity and perspectives from different HCPs disciplines. Together with findings from interviews of other stakeholders, these results will serve as important inputs in developing a complex intervention to improve diagnosis and evidence-based treatment of OA in persons with T2DM.

The complexity of diabetes care is well described [36] and diabetes HCPs indicated that they saw their role as addressing what they perceived to be immediate T2DM priorities, which included glycemic control and assessment of diabetes complications. While HCPs saw the impact of symptomatic knee OA on their patients with T2DM, particularly in limiting engagement in physical activity, there was no specific intention to screen for or address joint pain within diabetes visits. This is consistent with prior qualitative research which has showed that OA “doesn't make it to the top of the list” [21] for primary care physicians treating individuals with complex chronic conditions such as T2DM, being viewed as an inevitable consequence of aging and less important relative to other conditions [35], and reinforcing the concept of competing demands for health care providers [37]. Further, for endocrinologists and diabetes educators, addressing joint pain was not seen as their role within the health care system. While it is unlikely to be realistic or feasible for diabetes specialists to work up and manage OA, recognizing OA's importance as a diabetes comorbidity and understanding how to appropriately direct care may curb the adverse effects of untreated OA in persons with T2DM.

Participants who were endocrinologists and diabetes educators perceived insufficient knowledge and skills with respect to evaluation and management of knee OA. Prior research has shown this to be a barrier for primary care providers too [35], though we did not find this. Given the centrality of physical activity in T2DM management [38], and that knee OA is among the most important causes of walking difficulty [11], ways to empower HCPs to be able to screen for knee OA is important. Ideally, this would not require advanced knowledge or skills and could be efficiently done during a routine visit. Having accessible treatment pathways for OA may further motivate HCPs to assess joint symptoms. Participants in the current study perceived they had few treatment options to offer their patients; out-of-pockets costs precluded even physiotherapy for many. However, lack of awareness of resources may compound the issue. Most participants did not direct patients to the Arthritis Society, which in Ontario, Canada offers OA education and limited physiotherapy assessments without cost to the patient. Having comprehensive government-funded treatment programs for OA and ensuring HCPs are aware such programs has the potential to influence HCP behaviour.

While effective evidence-based treatments exist [17], the quality of OA care remains suboptimal. The biggest deficiency is in the provision of non-pharmacological therapies [18], including physical activity/exercise, weight management and education, which have greatest potential for benefit at least risk for individuals who also have T2DM. Participants in the current study generally did not feel it was their role to supervise physical activity for persons with OA, the most effective non-pharmacological therapy [39]. Given the dual importance of ensuring persons with T2DM can engage in regular physical activity [38], health systems should seek ways in particular for non-pharmacological OA therapies to be implemented widely.

Our study has many strengths. Using the TDF allowed us to comprehensively assess determinants of behaviour through a well-developed theoretical underpinning and common language. This will facilitate evidence-based links to behaviour change techniques [40] in the next phase of complex intervention development. We included HCPs across different disciplines, practice settings and years in practice to ensure we captured a wide variety of perspectives. While not all HCPs were expected to assume the same level of responsibility for musculoskeletal care, diabetes HCPs typically see their patients at frequent intervals and thus are in an important position to evaluate and direct care for issues that come up that influence T2DM self-management. Our sample size was similar to other studies that have assessed other HCP behaviours using the TDF [[41], [42], [43]]. Limitations of this study include less specificity of the target behaviour than in other TDF studies, and thus some important nuances to aspects of assessing and treating OA may have been missed. The majority of participants were from an urban setting (Toronto), recruited through investigators’ networks, and it is possible we failed to capture distinct perspectives from those practicing in rural and remote settings. As this study was conducted in Ontario, Canada, these findings may not transfer to other health care systems. Data to support including the domain “social influences” stemmed from a single participant, and while important, this should be confirmed in other studies. As comparison of behavioural determinants across different disciplines evolved in the analytic phases, this could be explored specifically in future studies.

This study identified behavioural determinants of assessing and treating knee OA in persons with T2DM from the perspectives of diabetes HCPs and included multiple domains of the TDF. This study, combined with perspectives of persons living with OA and T2DM, and well as those of other arthritis health professionals, is an important step towards developing an intervention to improve diagnosis and evidence-based treatment of knee OA in persons with T2DM to improve health outcomes.

## Author contributions

LKK and GAH conceived of the study. LKK, OK, EJW, CM, IS, JS, AW, NMI, LL, GAH, and JAP contributed to the study design. LKK and EJW contributed to data collection. LKK, OK, EJW, CM, IS, GAH, and JAP were involved in data analysis. LKK drafted the article with assistance from OK. All authors critically revised the article and approved the final version for submission. All authors had full access to all the data in the study. LKK, EJW, CM, IS, GAH directly accessed and verified the underlying data reported in the Article.

## Role of the funding source

This study was funded by a Project Grant, PJT-166069, from the Canadian Institutes of Health Research (CIHR). The funder had no role in the design and conduct of the study; collection, management, analysis, and interpretation of the data; preparation, review, or approval of the manuscript; or the decision to submit the manuscript for publication.

## Conflicts of interest

GAH has received research support as the Sir John and Lady Eaton Professor and Chair of Medicine, Department of Medicine, University of Toronto; LL receives salary support as the director of the Novo Nordisk Network for Healthy Populations, University of Toronto; all other authors declare no other relationships or activities that could appear to have influenced the submitted work.

## Data statement

The qualitative data generated and/or analyzed during the current study are not publicly available due to the Research Ethics Board-approved study protocol. However, the data can be made available from the corresponding author on reasonable request.
