# Supplementary material for: Barriers and enablers to health care providers assessment and treatment of knee osteoarthritis in persons with type 2 diabetes mellitus: A qualitative study using the Theoretical Domains Framework
Source: Osteoarthr Cartil Open. 2022 Aug 7;4(4):100299. doi: 10.1016/j.ocarto.2022.100299 (PMC9718241; doi:10.1016/j.ocarto.2022.100299)
Supplement: Multimedia component 1 [file mmc1.docx]

**Supplementary Table A. Theoretical Domains Framework domains and definitions used**

| **TDF Domain and Definition** | **Details** |
| --- | --- |
| **1. Knowledge**  An awareness of the existence of something | Knowledge (including knowledge of condition/scientific rationale): *An awareness of the existence of something*  Procedural knowledge: *Knowing how to do something*  Knowledge of task environment: *Knowledge of the social and material context in which a task is undertaken* |
| **2. Skills**  An ability or proficiency acquired through practice | Skills development: *The gradual acquisition or advancement through progressive stages of an ability or proficiency acquired through training and practice*  Competence: *One’s repertoire of skills, and ability especially as it is applied to a task or set of tasks*  Ability: *Competence or capacity to perform a physical or mental act. Ability may be either unlearned or acquired by education and practice*  Interpersonal skills: *An aptitude enabling a person to carry on effective relationships with others, such as an ability to cooperate, to assume appropriate social responsibilities or to exhibit adequate flexibility*  Practice: *Repetition of an act, behaviour, or series of activities, often to improve performance or acquire a skill*  Skills assessment: *A judgment of the quality, worth, importance. Level or value of an ability or proficiency acquired through training and practice* |
| **3. Social/professional role and identity**  A coherent set of behaviours and displayed personal qualities of an individual in a social or work setting | Professional identity: *The characteristics by which an individual is recognised relating to, connected with, or befitting a particular profession*  Professional role: *The behaviour considered appropriate for a particular kind of work or social position*  Social identity: *The set of behavioural or personal characteristics by which an individual is recognizable [and portrays] as a member of a social group*  Identity: *An individual’s sense of self defined by a) a set of physical and psychological characteristics that is not wholly shared with any other person and b) a range of social and interpersonal affiliations (e.g., ethnicity) and social roles.*  Professional boundaries: *The bounds or limits relating to or connected with a particular profession or calling*  Professional confidence: *an individual’s belief in his or her repertoire of skills and ability especially as it is applied to a task or set of tasks.*  Group identity: *the set of behavioural or personal characteristics by which an individual is recognizable [and portrays] as a member of a group*  Leadership: *The processes involved in leading others, including organising, directing, coordinating, and motivating their efforts toward achievement of certain group or organization goals*  Organizational commitment: *An employee’s dedication to an organisation and wish to remain part of it.* |
| **4. Beliefs about capabilities**  Acceptance of the truth, reality or validity about an ability, talent, or facility that a person can put to constructive use | Self-confidence: *Self-assurance or trust in one’s own abilities, capabilities, and judgement*  Perceived competence: *An individual’s belief in their ability to learn and execute skills*  Self-efficacy: *An individual’s capacity to act effectively to bring about desired results, as perceived by the individual* Perceived behavioural control*: an individual’s perception of the ease or difficulty of performing the behaviour of interest*  Beliefs: *The thing believed; the proposition/set of propositions held true*  Self-esteem: *The degree to which the qualities and characteristics contained in one’s self-concept are perceived to be positive*  Empowerment: *The promotion of the skills, knowledge, and confidence necessary to take great control of one’s life as in certain educational or social schemes; the delegation of increase decision-making powers to individuals or groups in a society or organization*  Professional confidence*: An individual’s beliefs in his or her repertoire of skills, and ability, especially as it is applied to a task or set of tasks.* |
| **5. Optimism**  The confidence that things will happen for the best or that desired goals will be attained | Optimism: *The attitude that outcomes will be positive, and that people’s wishes or aims will be ultimately fulfilled*  Pessimism: *The attitude that things will go wrong, and that people’s wishes or aims are unlikely to be fulfilled*  Unrealistic optimism: *the inert tendency for humans to over-rate their own abilities and chances of positive outcomes compared to those of other people* |
| **6. Beliefs about consequences**  Acceptance of the truth, reality, or validity about outcomes of a behaviour in a given situation | Beliefs: *The thing believed; the proposition or set of propositions held true*  Outcome expectancies: *Cognitive, emotional, behavioural, and affective outcomes that are assumed to be associated with future or intended behaviour. These assumed outcomes can either promote or inhibit future behaviours.*  Characteristics of outcome expectancies: *Characteristics of the cognitive, emotional and behavioural outcomes that individuals believe are associated with future or intended behaviours and that are believed to either promote or inhibit these behaviours. These include whether they are sanctions/rewards, proximal/distal, valued/not valued, probable/improbable. Salient/not salient, perceived risks or threats.*  Anticipated regret: *A sense of the potential negative consequences of a decision that influences the choice made: for example an individual may decide not to make an investment because of the feelings associated with an imagined loss*  Consequents: *An outcome behaviour in a given situation* |
| **7. Reinforcement**  Increasing the probability of a response by arranging a dependent relationship, or contingency, between the response and a given stimulus | Rewards (proximal/distal, valued/ not valued, probable/improbable): *Return or recompense made to, or received by a person contingent on some performance*  Incentives: *An external stimulus, such as condition or object, that enhances or serves as a motive for behaviour*  Punishment: *The process in which the relationship between as response and some stimulus or circumstance results in the response becoming less probable; a painful, unwanted or undesired event or circumstance imposed as a penalty on a wrongdoer*  Consequents: *An outcome of behaviour in a given situation*  Reinforcement: *A process in which the frequency of a response is increased by a dependent relationship or contingency with a stimulus*  Contingencies: *A conditional probabilistic relation between two events. Contingencies may be arranged via dependencies or they may emerge by accident*  Sanctions: *A punishment or other coercive measure, usually administered by a recognized authority, that is used to penalise and deter inappropriate or unauthorized actions* |
| **8. Intentions**  A conscious decision to perform a behaviour or a resolve to act in a certain way | Stability of intentions: *ability of one’s resolve to remain in spite of disturbing influences*  Stages of Change model*: A model that proposes that behaviour change is accomplished through five specific stages*  Transtheoretical model and stages of change: *a five-stage theory to explain changes in people’s health behaviour. It suggests that change takes time, that different interventions are effective at different stages, and that there are multiple outcomes occurring across the stages* |
| **9. Goals**  Mental representations of outcomes or end states that an individual wants to achieve | Goals (distal/proximal): *Desired state of affairs of a person or system, these may be closer (proximal) or further away (distal)*    Goal priority*: Order of importance or urgency of end state toward which one is striving*  Goal/target setting: *A process that establishes specific time based behavioural targets that are measurable, achievable and realistic*  Goals (autonomous/controlled): *The end state toward which one is striving: the purpose of an activity or endeavour. It can be identified by observing that a person ceases or changes their behaviour upon attaining this state; proficiency in a task to be achieved within a set period of time.*  Action planning: *The action or process of forming a plan regarding a thing to be done or a deed*  Implementation intention: *The plan that one creates in advance of when, where, and how one will enact a behaviour* |
| **10. Memory, attention, and decision processes**  The ability to retain information, focus selectively on aspects of the environment and choose between two or more alternatives | Memory: *The ability to retain information or a representation of a past experience, based on the mental processes of learning or encoding retention across some interval of time, and retrieval or reactivation of the memory; specific information of a specific task*  Attention*: A state of awareness in which the senses are focused selectively on aspects of the environment and the central nervous system is in a state of readiness to respond to stimuli*  Attention control: *The extent to which a person can concentrate on relevant cues and ignore all irrelevant cues in a given situation*  Decision making: *The cognitive process of choosing between two or more alternatives, ranging from the relatively clear-cut to the complex*  Cognitive overload/tiredness: *The situation in which the demands placed on a person by mental work are greater than a person’s mental abilities* |
| **11. Environmental context and resources** Any circumstance of a person’s situation or environment that discourages or encourages the development of skills and abilities, independence, social competence and adaptive behaviour | Environmental stressors: *External factors in the environment that cause stress*  *Resources/material resources: Commodities and human resources used in enacting a behaviour*  Organizational culture/climate: *A distinctive pattern of thought and behaviour shared by members of the same organization and reflected in their language, values, attitudes, beliefs and customs*  Salient events/critical incidents: *Occurrences that one judges to be distinctive, prominent or otherwise significant*  Person x environment interaction: *Interplay between the individual and their surroundings*  Barriers and facilitators: *In psychological contexts, barriers/facilitators are mental, emotional or behavioural limitations/strengths in individuals or groups* |
| **12. Social influences** Those interpersonal processes that can cause individuals to change their thoughts, feelings or behaviours | Social pressure: *the exertion of influence on a person or group by another person or group*  Social norms: *Socially determined consensual standards that indicate a) what behaviours are considered typical in a given context and b) what behaviours are considered proper in the context*  Group conformity: *The act of consciously maintaining a certain degree of similarity to those in your general social circles*  *Social comparisons: The process by which people evaluate their attitudes, abilities or performance relative to others*  Group norms: *Any behaviour, belief, attitude or emotional reaction held to be correct or acceptable by a given group in society*  Social support: *The apperception or provision of assistance or comfort to others, typically in order to help them cope with a variety of biological, psychological and social stressors. Support may arise from any interpersonal relationship in an individual’s social network, involving friends, neighbours, religious institutions, colleagues, caregivers of support groups*  Power: *The capacity to influence others, even when they try to resist this influence Intergroup conflict: Disagreement or confrontation between two or more groups and their members. This may involve physical violence, interpersonal discord, or psychological tension.*  Alienation: *Estrangement from one's social group; a deep-seated sense of dissatisfaction with one's personal experiences that can be a source of lack of trust in one's social or physical environment or in oneself; the experience of separation between thoughts and feelings*  Group identity: *the set of behavioural or personal characteristics by which an individual is recognizable [and portrays] as a member of a group*  Modeling: *In developmental psychology the process in which one or more individuals or other entities serve as examples (models) that a child will copy* |
| **13. Emotion**  A complex reaction pattern, involving experiential, behavioural, and physiological elements, but which the individual attempts to deal with a personally significant matter or event | Fear: *An intense emotion aroused by the detection of imminent threat, involving an immediate alarm reaction that mobilizes the organism by triggering a set of physiological changes*  Anxiety: *A mood state characterized by apprehension and somatic symptoms of tension in which an individual anticipates impending danger, catastrophe or misfortune.*  Affect: *An experience or feeling of emotion, ranging from suffering to elation, from the simplest to the most complex sensations of feelings, and from the most normal to the most pathological emotional reactions.*  Stress: *A state of physiological or psychological response to internal or external stressors*  Depression: *A mental state that presents with depressed mood, loss of interest or pleasure, feelings of guilt or low self- worth, disturbed sleep or appetite, low energy, and poor concentration*  Positive/negative affect: *the internal feeling/state that occurs when a goal has/has not been attained. A source of threat has/has not been avoided, or the individual is/is not satisfied with the present state of affairs*  Burn-out: *Physical, emotional or mental exhaustion, especially in one’s job or career, accompanied by decreased motivation, lowered performance and negative attitudes towards oneself and others* |
| **14. Behavioural regulation**  Anything aimed at managing or changing objectively observed or measured actions | Self-monitoring: *A method used in behavioural management in which individuals keep a record of their behaviour, especially in connection with efforts to changes or regulate the self; a personality trait reflecting an ability to modify one’s behaviour in response to a situation*  Breaking habit: *to discontinue a behaviour or sequence of behaviours that is automatically activated by relevant situational cues*  Action planning: *The action or process of forming a plan regarding a thing to be done or a deed.* |

*Structure and definitions guided by:*

Atkins L, Francis J, Islam R, et al. A guide to using the Theoretical Domains Framework of behaviour change to investigate implementation problems. *Implement Sci*. 2017;12(1):77. doi: 10.1186/s13012-017-0605-9

Cahill LS, Carey LM, Mak-Yuen Y, et al. Factors influencing allied health professionals' implementation of upper limb sensory rehabilitation for stroke survivors: a qualitative study to inform knowledge translation. *BMJ Open*. 2021;11(2):e042879. doi: 10.1136/bmjopen-2020-042879

Dobson F, Bennell KL, French SD, et al. Barriers and Facilitators to Exercise Participation in People with Hip and/or Knee Osteoarthritis: Synthesis of the Literature Using Behavior Change Theory. *Am J Phys Med Rehabil*. 2016;95(5):372-89. doi: 10.1097/PHM.0000000000000448

**Supplementary Table B. Semi-structured Interview Guide**

**Interview Guide: Physicians and Diabetes Educators**

*Thank you for agreeing to speak with me today. We’re in the first phase of a study that will develop and test an intervention to improve treatment of joint pain in people with diabetes but before we start the trial we really want to hear what things are like currently at your institution and your perspectives.*

*I’m going to start the interview by asking you a few questions in order to get to know you and your practice a little bit more. Then I’m going to ask you about the perspective about physical activity in people with diabetes, and in particular your experiences of caring for patients with diabetes who might also have joint pain or arthritis.*

*I have a list of questions to go through that serve as a guide only. I encourage you to talk about any aspect of the topic you wish. There are no right or wrong answers to these questions.*

*I will be audio recording the interview, in order to capture all the details of our conversation. This is a purely voluntary activity, so remember that you may end the interview at any time. If you need to take a break, please let me know and we can do so. Also, if you feel uncomfortable with any question, tell me and we can skip it. No personal information about you will be shared and you will not be identified to your colleagues or patients. No identifying information will be shared in any reports or publications. While we might use direct quotes in such reports, they will only be attributed more generally to someone who is a “physician”, etc. All the information provided by you will be kept strictly confidential and we hope you will feel able to speak freely as we genuinely want to hear your perspective.*

*If a question does not make sense, let me know so I can ask it more clearly. Take as much time as you like to answer the questions.*

*Do you have any questions before we begin?*

*(and if not, start* ***recording****)….*

1. Can you start by describing your current practice?

Probes:

- What does your typical day look like?
- How long have you been in your current practice setting?
- Are there other clinicians in your practice? If so, what are their roles?
- Tell me a bit about the range and types of patients you see.
- How much of your practice involves diabetes care?

1. Tell me about your experiences seeing patients who have diabetes…just very generally, what are the things that you consider the priorities to cover in a routine follow up visit?

Probes:

- [EC/R] Typically, how long are the appointments?
- [I] Recognizing that each clinical encounter is really busy, how to you decide what you can get to and what you can’t.

1. So the prevalence of multimorbidity is increasing. How do you approach the management of patients with diabetes who also have other chronic conditions – how do you juggle this? How do you think it affects your patients with diabetes?

Probes:

- Are some other conditions more top of mind, or given more consideration, than others? Which ones and why? Can you give me any examples?

1. When it comes to physical activity and being active, do you have a usual approach to talking about this with patients with diabetes? Can you give me some examples?

Probes:

- [M/A/D] Do you tend to bring up physical activity with all of your patients or do you wait for patients to initiate the discussion?
- [I] How top of mind is it? [BR] Do you have any tricks or tools you use to remind yourself to ask about physical activity in your patients with diabetes?
- [M/A/D] When someone is not active, does that prompt any further steps?
- [K] Do you ever refer to any guidelines or references? If so, which ones?

1. So, MSK problems and arthritis pain is very common in the general population. Does joint pain ever come up in your discussions with your diabetes patients?

Probes:

- How frequently do you have these kinds of conversations with your diabetes patients? What usually prompts the discussion?
- What kind of impact do you think joint pain has on their life? Their diabetes management?

1. Do your patients tend to seek health care with you or others specifically to manage their joint pains?

- [S/PR/I] How does assessment of their joint pain fit within your professional role or responsibility?
- [S] How confident are you that you can assess a patient’s joint if they tell you they have joint pain? Tell me about a situation when you had to do a joint assessment?
- [K, S] Tell me about any training you’ve received about musculoskeletal conditions, both diagnosing and treating them.
- [K] Do you refer patients who report joint pain or arthritis to see any other health care practitioners? Who do you refer them to?
- [O] Do you think addressing their arthritis will make a difference for your patients?

1. When it comes to helping patients with diabetes manage their joint pain, do you tend to recommend any specific therapies for them? What have you found helpful for these patients?

Probes:

- [ECR, K] Are there any practitioners or programs that you refer to, to help with treatment? Does physiotherapy ever come up? What about other forms of therapy?
- [K, S] Have you recommended any medications to manage pain?

1. I want to know about your thoughts on physical activity for patients living with *both* diabetes and joint pain or arthritis, especially in weight-bearing joints like the knee. How do you currently discuss physical activity with these patients? Do your recommendations change at all when they have both conditions? If so, how?

Probes:

- [K[ What informs your recommendations in the context of joint pain?
- [ECR] Do you have any resources available to you that are helpful?
- [SI] Do you have any colleagues who are helpful when exploring physical activity with your patients?
- [BAC] What do you see as the downsides of asking about and recommending physical activity to your diabetes patients who also have joint pain?

1. What kind of things, if any, would help you manage patients with both diabetes and joint pain or arthritis?

Probes:

- Are there any resources that would be helpful for you?
- What would help you make recommendations or referrals for physical activity for these patients? Please give me an example.
- Have you used toolkits or other reminder systems in the past? What was your experience like?

1. Is there anything else that you would like to comment on that I haven’t asked you about today?
2. I would like to finish with briefly asking a few demographic questions which we’re asking all our participants: What is your...

| Age | 20-29, 30-39, 40-49, 50-59, 60-69, 70+ |
| --- | --- |
| Gender | Man, Woman, Other, Prefer not to answer |
| Professional designation | Family physician, endocrinologist, diabetes educator |
| Number of years in practice | 0-4, 5-9, 10-14, 15-19, 20-24, 25-29, 30+ |
| Location of practice | Urban, suburban, rural |
| Is University affiliated practice? | Yes, no |

Thank you very much for your time and the information you shared today.
